# Understanding Parent Perspectives Concerning Adolescents’ Online Access to Personal Health Information

**Published:** 2016-03-14

**Authors:** Gregory L. Gaskin, Janine Bruce, Arash Anoshiravani

**Affiliations:** a Department of Pediatrics, Stanford University School of Medicine, Stanford, CA

## Abstract

**Background:**

Although today's youth are interested in using the internet to access and manage information related to their health, little information exists about parental attitudes towards the release of health information to adolescents.

**Methods:**

Structured interviews were conducted with the parents of 83 adolescents detained at a large Northern California juvenile detention facility to examine parental perceptions toward allowing their children online access to their own health information.

**Results:**

The majority of parents interviewed (70%) wanted their children to have online access to their own health information. Seventy-nine percent of these parents were also comfortable allowing their children to choose with whom they would share this information.

**Conclusions:**

This study is one of the first to examine parental attitudes towards providing adolescents access to their own health information, and the first among parents of underserved youth. This study demonstrates that parents may be quite supportive of allowing their adolescent children to have secure online access to their own health information.

## Introduction

The published literature has identified numerous benefits and health impacts (potential and realized) of patient portals and/or personal health records for pediatric populations.[1-3] Allowing patients access to their own personal health information (PHI) has been documented to increase patient engagement and empowerment, improve health literacy, improve patient safety, and provide more timely patient-care team communication.[4-7] Although slowly changing, most existing pediatric patient portals focus on enabling parental access to their child's health information.[8]

However, lost among the focus on young children and their caregivers, remains the population currently most comfortable and engaged with technology: adolescents. Today's adolescents have demonstrated a high level of interest in using the internet to access and manage information related to their health;[9-11] however, little work has been done to directly assess the opinions of these youths and their families. Some studies have raised questions about giving adolescents access to their own PHI, mostly focusing on the complicated legal issues, perceived risks, or technical details of implementation.[12-15]

Few data exist about the use of patient portals and online personal health records by adolescent patients and their families. Despite recommendations from both the American Academy of Pediatrics and the Society for Adolescent Health and Medicine for inclusion of online access to PHI for adolescents as a part of high-quality adolescent health care,[12,16] to date very few studies have examined the preferences of adolescents themselves[11,17] and only one the concerns of the parents of adolescents with regard to PHI accessible through the internet.[11] Especially little work has been done to explore the role that health information technology (HIT) can play to improve the provision of health care for underserved youth. Underserved populations, such as detained youth, have greater unmet health needs and more fragmented healthcare than the general adolescent population.[18,19] The existing gap in HIT knowledge and innovation only serves to intensify the existing health disparities and fragmented care faced by these young people.

This paper aims to assess parents' attitudes toward, perspectives on, and concerns about allowing adolescents access to their own personal health information online. In particular, this work focuses on the parents of a particularly underserved and high-risk population of youth. We hypothesize that if utility and parental acceptance can be found in a population perceived to be particularly challenging, it will lay the foundation for challenging the commonly-held assumptions that parents are uncomfortable with their adolescent children having access to their own health information, especially among populations with healthier and more trusting parent-child relationships.

## Patients and Methods

We conducted structured telephone interviews with the parents/guardians of 13-18 year-old adolescents detained at a large Northern California juvenile detention facility. The interviews were designed to examine parental perceptions of allowing their children online access to their own PHI.

The interviews lasted approximately 10 minutes, were conducted in English and consisted of both quantitative and qualitative questions. In total, we attempted to contact the parents/guardians of 305 randomly selected youths, of which we spoke with 159, 83 of whom agreed to participate in the interview and spoke English comfortably.

Fisher's Exact test[20] was used to examine differences in rates of parental acceptance of youth's online access to PHI by race, gender and age of the youth and the relationship of the respondent to the youth. Qualitative data were examined using both summative content analysis[21] and theme analysis to describe parental concerns about allowing online access to their child's health information. Study authors GG and JB collaborated to inductively co-code the data and develop a codebook through a consensus-building process until negotiated intercoder agreement was achieved.[22,23] Identified themes were presented to AA for feedback based on his expertise in the field of adolescent health. Through an iterative process, all three authors met multiple times to refine the themes and identify representative quotes.[24,25] All methods in this study were approved by the Stanford University and Santa Clara Valley Medical Center Institutional Review Boards.

## Results

The children of parents surveyed were predominately male (88%) and Hispanic (70%) with a median age of 16 years (Table 1). These demographics are representative of the population of adolescents detained at the study facility and nearly identical to previously published studies in this specific setting.[26,27] The majority of parents/guardians interviewed (70%) wanted their child to have secure, online access to their own PHI. There were no statistically significant differences between the proportion of parents who wanted to give their child access based on the gender, age or race/ethnicity of the child or the relationship of the child to the respondent. Parents/guardians who reported ever having an incident in which they were missing necessary health information for themselves or their children were unanimously in favor of giving their child online access to their health information versus 65% of parents who had never experienced such missing information who were in favor of such a system (p=0.004).

Among the 70% of parents/guardians who were in favor of allowing their child to have access to their health information, 100% believed that the adolescents should have the ability to share this information with their parent/guardians through the online system. A large majority (79%) of these parents/guardians was also comfortable leaving the choice with their children, allowing them to choose with whom they would share their health information.

Sixty-three percent of respondents expressed no concerns about allowing their child online access to their PHI. A qualitative content analysis of the comments from the 31 parents/guardians who did express concerns revealed three major areas of concern that included 1) security concerns related to a malicious third-party hacking their child's online account to gain illegal access to their PHI, 2) issues regarding authorized access to the their child's health information, and 3) perceived barriers to utilization (Table 2).

## Discussion

This study is one of the first to examine parental attitudes towards providing adolescents access to their own PHI and the first to examine such attitudes among an underserved population of youth. Whereas other work in this field has focused on the legal and technical challenges inherent in allowing teenagers to have access to their own PHI,[12-15] this study elicited parents' actual thoughts and concerns. We demonstrate that even among a population of youth that tends to have very conflicted relationships with their parents,[28] there is a high level of parental interest in allowing their adolescent children access to and control of their own health information online. In contrast to published policy statements [12,16] and the small amount of existing research,[17] the majority of parents in our sample were comfortable with allowing their children to see their health information, and had no concerns about their children choosing which, if any information they would share with them. The concerns parents did express tended to center around the privacy of health information and could likely be adequately addressed through education about the privacy and security features of the specific patient portal or personal health record being used.

We acknowledge several limitations regarding this work. The study was conducted among the parents of a very specific, high-risk adolescent patient population. Differences in our findings from previous work may represent either shifting parental perspectives over time or differing views among different populations. Additionally, since this is a small pilot project with interviews conducted only in English, the results may not be generalizable to a broader population of youth and their families. Larger samples in more diverse healthcare settings will further contribute to our understanding of parents' attitudes toward allowing their adolescent children online access to their own personal health information.

## Conclusion

As health care organizations begin to implement patient portals for adolescent patients, it is vital to understand the perspectives and concerns of both adolescents and their parents toward such systems. This study is the first to demonstrate that parents may be quite supportive of allowing their adolescent children to have online access to and full control of their own PHI. Parents' primary concerns might be alleviated by using a portal with robust privacy and security features. Taken with previous work demonstrating adolescents' interest in accessing their own PHI,[11,17] this study suggests that continued efforts to implement online-accessible records for adolescents might be feasible and useful.

## Figures and Tables

**Table 1:** Demographic Information and Parental attitudes toward allowing their child online access to their own health information

Demographic Characteristics	N	(%)	p-value^a^
Gender of Child:			
Male	73	88	
Female	10	12	
Relationship of Respondent to Child			
Mother	65	78	
Father	9	11	
Other Female Guardian	9	11	
Race of Child:			
Hispanic	58	70	
African-American	12	15	
Caucasian/White	9	11	
Other/Unknown	3	4	
Age of Child			
13	4	5	
14	11	13	
15	12	14	
16	23	28	
17	27	33	
18	6	7	
**Do you want your child to have online access to their own health information?**			
Yes	58	(70)	
No	25	(30)	
Gender of Child:			
Male	54/73	(74)	
Female	4/10	(40)	p = 0.06
Relationship of Respondent to Child			
Mother	47/65	(72)	
Father	7/9	(78)	p = 0.24
Other Female Guardian	4/9	(44)	
Race of Child:			
Hispanic	41/58	(71)	
African-American	8/12	(62)	p = 0.88
Caucasian/White	7/9	(78)	
Other/Unknown	2/3	(67)	
Age of Child			
13	3/4	(75)	
14	9/11	(82)	
15	5/12	(42)	p = 0.35
16	16/23	(70)	
17	20/27	(74)	
18	5/6	(83)	

a By Fisher's Exact Test

**Table 2:** Themes and representative quotations surrounding parental concerns about allowing adolescents access to their own personal health information from the 37% of parents/guardians who expressed having any concerns about such access

Domain Theme	Quotes
**D1. Security Concerns**	

T1. Risk of illegal hacking or malicious breach of online account	*“As long as nobody hacks into it”*
	*“Just on how secure it will be…identity theft”*
	*“I've been hacked before…[I am] worried about [the] privacy of information”*
	*“[Make sure it's a] site that hackers couldn't easily get into”*
	*“How do you prevent hackers or any unauthorized use of the system?”*
	*“People can get access to passwords and usernames”*

**D2. Access Issues**	

T2. Ability of the general public to access private information through the internet	*_“Other people can get into it and find a lot of information…anybody could get into it and use it”*
	*“[I] don't know who's capable of seeing this kind of information”*
	*“[E]verybody could get a hold of what's online”*
	*“If it is open to the public”*
	*“Their health information being exposed to the public”*
	*“[If there were] some type of glitch…I would hate for [this information] to get out”*
	*“[Make sure] not everyone has access to it”*

T3. Ability of adults involved in child's life who do not need to see this information to have access	*“Anybody and everybody who is [a] government [employee] can access it”*
	*“Maybe he doesn't want to share that information with anybody…[he] might be intimidated to share information with probation officers”*

T4. Necessity that access to information should be controlled by the parent or doctor	*“[I] only want information coming from their doctor”*
	*“Because he's a minor, I probably don't want him to access it. I would like access to it, but he doesn't need it. If he was 18, I could see why he would need access to that. But right now, I still schedule his doctor's appointments.”*

**D3. Perceived Barriers to Utilization**	

T5. Perceived immaturity of teen makes it inappropriate to allow them access to their health information	*“[C]hildren might not be able to handle it”*
	*“They have to recognize that they shouldn't share this just because they think somebody is cool or they're friends”*
	*“It's kind of scary…[having access to this information] would just put him into a downward spiral situation…so many children are insecure”*
	*“They are minors…and minors aren't always aware”*
	*“They're in juvenile hall, so that says a lot about their immaturity”*
	*“They don't know who to share that information with”*
	*“They need to have this access when they are the right age and can make the right decisions”*

T6. Parental discomfort with using the internet to store health information	*“I'm not really good at being online.”*
	*“Only papers and fax are acceptable”*
	*“I don't like putting my information on there [the internet], but that's the thing right now.”*
	*“In the news, all of this is online and everybody could get a hold of what's online”*
	*“[I] don't like personal business on the internet”*
	*“The fact…that it is online”*
